# Cocaine in Urethral Surgery

**Published:** 1888-01

**Authors:** A. F. Sampson

**Affiliations:** Galveston, Texas


DANIEL'S

### TEXAS MEDICAL JOURNAL.

PUBLISHED MONTHLY.—SUBSCRIPTION $2.00 A YEAR.

Vol. 3. AUSTIN, JANUARY, 1888. No. 7.

By unanimous vote of the members, this Journal was made the Official Organ of the Austin District Medical Society.

### ORIGINAL ARTICLES.

CONTRIBUTED EXCLUSIVELY TO THIS JOURNAL.

*The Articles in this Department are accepted and published with the understanding that we are not responsible for, nor do we indorse the views and opinions of the writers by so doing.*

### COCAINE IN URETHRAL SURGERY.

*By A. F. Sampson, M. D., Galveston, Texas.*

For Daniel's Texas Medical Journal.

THE recent advancements in Urethral Surgery are so replete that it seems but little remains to be added. But recently the Otis method for the radical cure of Urethral Strictures has so forcibly demonstrated its claims that no surgeon now fails to appreciate the value of this generous investigator's contributions to science. And as an adjunct to the treatment of Urethral Strictures Newman's Electrolytic System should not be ignored.

Upon careful consideration of the following observations we can not fail to appreciate the fact that cocaine anæsthesia is destined to play an important part in Urethral Surgery. In the spring of 1885 I became convinced that cocaine was the anæsthetic, *par excellence*, in urethral surgery, and have since studied its effects, being assured that by its employment quite an advantage would be gained in this special branch.

Before entering upon the treatment of any case there is nothing that fortifies the surgeon more than a clear diagnosis with a full comprehension of the existing pathological condition. In gaining these advantages we must commend cocaine anæsthesia loudly.

A case of suspected urethral stricture is presented; the history is taken, parts examined casually, the proper examination of the urine had. Now it remains to devise our diagnosis and pathological changes, which not only necessitates an exploration of the canal with the proper instruments, but also an inspection must be had to fully reach our conclusions.

The exquisitively sensitive condition of the urethra that is invariably found to exist with urethral stricture precludes the possibility of making a satisfactory exploration, unless an anæsthetic be used, or we recklessly defy shock, or see the patient for the last time, who justly should become indignant at the unnecessary amount of suffering inflicted. The advantage of cocaine over general anæsthesia to gain the diagnosis and pathology, both to physician and patient, is too patent to give in detail. But the very fact that a patient can be fortified by the knowledge that he will undergo a thorough investigation without pain, the dangers of urethral shock, or be deterred from pursuing immediately his avocation, and without any of the dangers of general anæsthesia, and that he will be thoroughly conscious of all that is going on, must serve to produce a mental effect that affords the surgeon no little advantage.

I have taken special pains in observing the effects of cocaine in preventing *urethral shock.* By the term urethral shock, I would be understood as referring to the constitutional disturbance that we have found to follow the introduction of instruments in the urethral canal; manifested by rigors with its sequellæ, a condition that we have learned to dread in urethral surgery. Herein cocaine has never disappointed my most sanguine expectations.

Within the last three years, under the anæsthetic influence of cocaine, I have performed seventy-four internal urethrotomys for the radical cure of strictures, besides frequently introducing sounds and the urethra metre without encountering a single case of urethral shock. This experience must establish a claim for cocaine that will command special attention. I hold that ere long, he who would have to make an exploration of either the urethra or bladder, without first gaining the anæsthetic effects of cocaine, would be regarded culpable of great neglect should shock occur.

I cite the following case to illustrate the properties of cocaine in preventing urethral shock: Mr. J. applied for treatment of an obstinate gleet, that I thought due to a stricture. My preparations were made for a retro-injection, when the patient remonstrated, stating that if I introduced the instrument he would surely have a severe chill, etc., from which he nearly lost his life in a former treatment. I prevailed upon him to permit me to carry out my purpose, as the instrument was not of sufficient size to put the urethra on a stretch, it being a tube of only 16 m. m. circumference, nor was the injecting fluid irritating. A few hours after, much to my chagrin, I was summoned to my patient to find him with a temperature of 105° F., and a history of a severe rigor. *I had not used cocaine.* The gravity of this disturbance from so slight a cause awakened in me a keen appreciation for cocaine in controlling urethral shock. Having regained the confidence of this patient, who, fortunately, was a gentleman of intelligence, by assuring him that I had a means of preventing the much dreaded shock, I administered an injection of ten per cent of cocaine, and under its influence made a full and satisfactory exploration of his urethra without any manifestation of shock recurring. Here we see a case predisposed to shock suffer severely from an inconsiderate interference, whereas, under cocaine a much more extended and repeated exploration of urethra produced no untoward symptoms.

Again, an interesting case of reflex neuroses, due to urethral irritation, that simulated intermittent malarial fever, reported in the last November number of the *New York Medical Record*, the use of cocaine not only contributed to the diagnosis, but also manifested its powers in preventing urethral shock in a case markedly predisposed. My first examination without, resulted in a constitutional disturbance, whereas, under cocaine, the urethra metre was used and internal urethrotomy performed. No disturbance again.

The antiphlogistic properties of cocaine is another impression of this drug that should influence our preference for this anæsthetic in urethral surgery.

That internal urethrotony has been, to a marked degree, simplified, and this operation enhanced by its use, both to the surgeon and patient, does not admit of cavil. The majority of my operations were made without the aid of a single assistant. Owing to the hæmostatic effects of *the drug* the hemorrhages following the incisions have proven but slight. Patients experience so little disturbance from an internal urethrotomy under cocaine, that it is difficult for them to realize that an operation has been performed of any consequence. With most of my cases I have not found it necessary to confine them to bed.

The method adopted in gaining cocaine anæsthesia in the urethra: First, irrigate with about one quart of warm antiseptic solution; this seems to cleanse the canal of all mucus, besides gaining the antiseptic advantages 'ere incising a stricture. Should the introduction of the irrigating tube produce pain, it is safe to inject a solution of cocaine as the first step. Then the irrigation is followed by an injection of about twenty minims of a ten per cent. solution of cocaine, retained in the canal about fifteen minutes, which is generally found time sufficient to gain the desired effects. Should there be any sensibility still existing I renew the cocaine injection. By this method I have never failed to gain satisfactory anæsthesia. The nature of the mucous membrane of the urethra seems to contribute to the susceptibility of these parts to the influence of the drug. When there is a state of granular urethritis existing, that is clearly manifested by an inspection, the patient is instructed to inject a ten per cent solution mur. cocaine on voiding his urine during the following twenty-four hours; this insures freedom from pain that has been found to be so severe that the party loathed further interference.

A very unique case wherein cocaine played an important part in deriving the diagnosis, pathology and successful treatment I wish to add. Mr. M. consulted me in September, 1887. His history and examination of urine would have led me to suspect urethral stricture. Under cocaine the urethra meter gave negative results. My urethral speculum was then introduced, and by the aid of a hand mirror a thorough inspection of the first four inches of the canal gained. This plainly revealed the cause of the disturbance in a small wart growing from the superior surface, two inches from meatus, that by its presence had established a granular urethritis. The cocaine anæsthesia being perfect, it was an easy matter through the fenestrum of the speculum to remove the growth with a Wilde's snare, and treat the urethritis. This treatment accomplished all that could be desired.

Much satisfactory information is to be gained by ocular inspection of the urethra, which can easily be done by means of the fenestrated bivalve urethral speculum that I devised two years since. I claim this as a recent advancement in urethral surgery of some importance, wherein cocaine anæsthesia aids us. Upon this point Prof. F. N. Otis has written that valuable aid has been gained in his recent investigations by a satisfactory inspection of the urethra.

In making a resume of the claims that cocaine has made in the advancement of urethral surgery, we find:

1. Its psychical effects.

2. Its valuable aid in obtaining a clear diagnosis and the true pathology.

3. Its prevention of urethral shock.

4. Its antiphlogistic effects.

5. Its hæmostatic effects.

6. The great degree that it has simplified internal urethrotomy.

7. Enabling an easy and satisfactory inspection of the urethra throughout its first four inches.

